# From hot tub to high altitude: The makings of a human physiology field expedition and the next generation of physiologists

**DOI:** 10.1113/EP094074

**Published:** 2026-07-06

**Authors:** M. M. Tymko, K. J. Smith, A. R. Bain, I. A. Fernandes, B. S. Stacey

**Affiliations:** ^1^ Integrative Cerebrovascular and Environmental Physiology SB Laboratory, Department of Human Health Sciences University of Guelph Guelph Ontario Canada; ^2^ Cerebrovascular Health, Exercise, and Environmental Research Sciences Laboratory, Department of Exercise Science, Physical and Health Education, Faculty of Health University of Victoria Victoria British Columbia Canada; ^3^ Integrative Vascular Physiology Lab, Department of Kinesiology University of Windsor Windsor Ontario Canada; ^4^ Human Neurovascular Control Laboratory Department of Health and Kinesiology Purdue University West Lafayette Indiana USA; ^5^ Neurovascular Research Laboratory Faculty of Life Sciences and Education University of South Wales Pontypridd UK

## 1 INTRODUCTION

Long before Nobel Prize‐winning discoveries in digestive physiology and cellular oxygen sensing by Ivan Pavlov, William Kaelin Jr, Peter Ratcliffe and Gregg Semenza, pioneering integrative physiologists laid the foundation for modern environmental and high‐altitude physiology. In 1878, Paul Bert published *La Pression Barométrique*, helping establish that reduced oxygen partial pressure is the primary mechanism underlying high‐altitude mountain sickness (Bert, 1878). In the late 1800s to early 1900s, Nathan Zuntz helped develop methodologies crucial for high‐altitude integrative physiology, including respiratory apparatus, metabolic measurements, exercise testing and portable gas analysis systems during field expeditions. During the same epoch, while helping to establish the Capanna Regina Margherita on Monte Rosa (4559 m), Angelo Mosso performed some of the first high‐altitude integrative physiology experiments examining how this environment influences the brain, circulation, fatigue and human performance (Di Giulio & West, 2013; Mosso, 1897; Zuntz et al., 1906). Both Zuntz and Mosso were not just pioneers in establishing physiology in mountain frontiers but also laid the intellectual and methodological groundwork for future researchers and trainees to explore the human capacity in extreme environments. Zuntz's influence is especially important, as his later expeditions included luminaries like John Haldane and Joseph Barcroft, which inspired researchers like Mabel Purefoy Fitzgerald, August and Marie Krogh, Griffith Pugh, John West, Charles Houston, Peter Bärtsch and John Severinghaus (highlighted in West, 1998). The importance of this early work is not solely based on the research performed, but the training environments and systems that these prominent scientists developed. Like the research itself, these expeditionary accounts inspire us and lay the foundation for a new generation of researchers to carry this important work forward.

Throughout this special call on ‘Integrative Human Physiology in Hypoxia’, a central theme of the featured studies draws on field‐based research spanning more than a century of integrative high‐altitude physiological research. Many of these studies are conducted in authentic hypoxic environments, providing a unique and indispensable window into integrative human physiology. Like the early field‐based altitude research, and much like the unseen work behind a film set, many of the logistical, technical and human elements underpinning field‐based high‐altitude research go unnoticed. Field‐based physiological research is special because, by its very nature, it is messy, creative, collaborative, and occasionally held together by Transpore tape, coffee and optimism. It requires interdisciplinary thinking, persistence, creative problem‐solving, and, importantly, the ability to assemble a cohesive team that can function on little sleep and low oxygen. In this editorial, we offer an insider perspective on the realities and experiences of conducting high‐altitude field research, drawing on our 2025 expedition to the Barcroft Field Station (3800 m), California, USA. We also highlight the benefits of field research that expand beyond the physiology learned – that is, trainee experiences, networking and (usually, but not always) making science fun.

On this trip, the team consisted of 44 co‐investigators made up of undergraduate and graduate trainees, PIs and clinicians, as well as an alarming amount of laughter amongst colleagues turned friends. Indeed, field research provides an opportunity to build local and international collaborations, while offering a truly unique training environment for highly qualified personnel (HQP). Upon reflection, one of the most meaningful outcomes of our recent high‐altitude expedition was not just the data we collected (~18 independent studies worth!), but what the experience could provide for our trainees. Field research gives students something that cannot be easily recreated in a classroom or controlled laboratory: the chance to troubleshoot in real time, adapt under pressure, work as part of a large scientific team and experience the strange joy of doing high‐quality research in a place where simply walking up a hill feels like stage 4 of the Bruce protocol.

A question that is often asked following these amazing journeys is, How did the expedition come to fruition? Sometimes, the best ideas happen when you least expect them. It was March 2024, and a few of us, Smith, Bain and Tymko, were attending the Okanagan Cardiorespiratory Symposium (OKCRS), held biannually at SilverStar Ski Resort (Vernon, BC, Canada). This meeting was recently highlighted by *Experimental Physiology*’s Editor‐in‐Chief, Prof. Damian Bailey (Bailey, 2026). One of the unwritten requirements of attending this conference, at least in our interpretation of the scientific programme, was that a hot tub must always be within arm's reach to ensure our cardiovascular health was being optimally maintained through intermittent heat exposure, in between science and night skiing sessions. Somewhere between snow‐covered slopes, pruned fingers and mildly dehydrated brains, we floated the idea of co‐leading a high‐altitude research expedition to the Barcroft Research Station in the White Mountains of California.

Each of the expedition PIs has benefited enormously from international training experiences, which we consider critical to our development as researchers. We have been fortunate to conduct field work in Nepal (Smith, Tymko), Peru (Bain, Tymko and Stacey), Croatia (Bain and Tymko), and at the United States (Smith, Bain and Tymko). These experiences shaped all of us both personally and professionally, and we would likely not be in our current faculty positions without them. They taught us how to lead, collaborate, improvise, fail productively, persevere and occasionally carry very expensive equipment through places where equipment was clearly never meant to be carried.

Just as importantly, these experiences showed us the value of giving trainees their own international field‐research opportunities. For HQP, these expeditions are not simply ‘cool trips’. They are immersive training environments where students learn technical skills, leadership, teamwork, participant care, protocol management and scientific resilience. In many ways, our goal was to recreate for our students the kinds of formative experiences that had shaped us, and those that came before us.

What started as an off‐the‐cuff conversation, half joke and half dream, could have easily faded into the graveyard of good ideas that never make it out of the hot tub time machine. Despite concerns of funding and, at times, logistical nightmares, we remained determined. Tymko gave the idea a gentle shove forward, past the point of no return, by officially booking the Barcroft Research Station for August 2025 (14 months away). Once the intention was formalized, it was game on.

After returning home from the conference, it was not long before Tymko reached out to his close friend and colleague across the pond, Dr Benjamin Stacey, then a relatively new faculty member at the University of South Wales. Stacey had been part of our 2018 high‐altitude expedition to Peru (Tymko et al., 2020). Like a true biophysiology expeditionary navigator (Ben), Stacey was well versed in nearly every laboratory skill required for this kind of work, including cerebrovascular ultrasound, blood collection and analysis, and cardiorespiratory monitoring. In other words, he was exactly the kind of person you want nearby when the oxygen is low, the schedule is full and something expensive starts beeping at you.

One of the most rewarding aspects of international fieldwork is the way professional relationships become personal ones. A perfect example is how Dr Igor Fernandes became involved in the project. After booking the research station, Tymko and his family embarked on a road trip from southern Ontario to visit relatives in Western Canada, zig‐zagging across the United States along the way. One stop was particularly memorable: Lafayette, Indiana, where Tymko met Fernandes, then a newly appointed Assistant Professor at Purdue University. Fernandes's previous research and expertise in autonomic assessment and lab‐based hypoxia was well known to the team, and we were excited to try and recruit him for the White Mountain: Summer 2025 (WM‐S25) research expedition.

Fernandes was temporarily storing ultrasound units that Tymko had impulsively purchased on eBay, and which would eventually become critical pieces of equipment for the Barcroft studies. Each of us had long admired Fernandes's work from his time in Brazil, and the conversations with Fernandes flowed naturally. Somewhere between catching up, talking science and probably wondering whether buying ultrasound machines on eBay was a sensible long‐term career strategy, the idea emerged to push the cerebrovascular physiology field further by using stellate ganglion block (SGB) as an experimental tool. Fernandes's excitement about the expedition was infectious, and by the end of the visit, there was an open invitation for him to join us at Barcroft.

Fast‐forward to our preparations. We decided to host the sea‐level portion of the expedition through Tymko's home institution, the University of Guelph, which offered centrality, space and infrastructure. Much of early 2025 was spent preparing our graduate students for the proposed research projects at the home institutions of Bain (University of Windsor), Smith (University of Victoria), Stacey (University of South Wales) and Fernandes (Purdue University). In total 18 primary and sub‐studies were piloted before arriving in Guelph. This meant training students on different experimental techniques, developing and refining appropriate analysis procedures, building confidence across teams, and mentally preparing everyone for the whirlwind of data collection that was about to unfold over a short but intense 2‐week window at Guelph in late May and early June. While preparations were underway at each institution, trainees began contacting their soon to be expedition teammates through Zoom meetings. Many unaware of the positive impact and close relationships that would soon form during the immersive and close quarter research they were about to embark on. This is perhaps the most valuable component of field‐based expedition research. No single laboratory experiment can accomplish what these expedition‐based studies can. By working alongside peers from other institutions, disciplines and scientific traditions, trainees learn that discovery is not produced solely by individual expertise, but by shared problem‐solving, trust, adaptability and collective purpose in a challenging and dynamic environment. In this sense, field‐based research does more than generate meaningful and rich data; it helps build the next generation of integrative physiologists.

Thus, the preparation phase for baseline testing was an HQP training boot camp in the best possible sense. Students learned ultrasound, cardiorespiratory monitoring, blood sampling, participant management, data collection logistics, and, perhaps most importantly, how to work within a large, interdisciplinary research team. They also learned that ‘field work’ begins long before anyone reaches the field station. It begins with packing lists, ethics approvals (University of Guelph REB #1487), equipment checks, dry runs, caffeine, troubleshooting and the slow realization that no matter how carefully you plan, something may go sideways… but we were prepared.

We successfully completed our baseline sea‐level experiments at the University of Guelph by mid‐June 2025. In July, our convoy of researchers and students assembled in Las Vegas, lugging equipment, consumables, and enough ultrasound gel and saline to fill a bathtub. From there, we braced ourselves for the long drive, which involved the notorious 1.5‐h bone‐rattling dirt road to Barcroft. The four large vehicles we rented groaned with every bump, and those of us driving silently apologized to the students in the back seats, who were absorbing most of the punishment. In tandem, we were met by Dr Travis Gibbons (Northern Arizona University), a colleague well acquainted with field‐based research at the Barcroft Lab, who made the journey from Flagstaff in a ground hugging two‐door coupé carrying a large dewar to freeze, store and transport blood samples as well as nearly half of his lab equipment to aid our research.

Arriving at the station felt like stepping into another world. Steven Devanzo, station manager, chef extraordinaire and steady presence at the Barcroft lab, welcomed us with hearty meals and calm reassurance as we settled into life at 3800 m. While we were unpacking and setting up three dedicated research labs preparing for night 1 of testing, for many of us, altitude illness struck quickly: headaches, nausea, restless sleep and the classic signs of acute mountain sickness. At altitude, oxygen pressure is markedly lower, forcing the body to hyperventilate to maintain oxygen saturation. This comes at the cost of respiratory alkalosis, changes in cerebral blood flow and many restless nights filled with periodic breathing. For scientists studying these physiological adaptations, the line between participant and researcher can become blurred. We were studying the effects of hypoxia while actively being humbled by hypoxia. The PIs on this trip all had prior experience at altitudes above 3800 m, except for Fernandes. However, for nearly all our trainees, more than 25 graduate students, this was their first exposure to this type of field research environment.

Fortunately, we had several clinicians on site to ensure that anyone experiencing altitude sickness was monitored closely. Trainees, many of whom were interested in pursuing medicine as a career, had the opportunity to work in close proximity to these clinicians as they performed many invasive research procedures and monitored the health of the team. Importantly, our students were able to witness, in real time, the ethical and practical responsibilities of conducting human research in a challenging environment. They saw that good field physiology is not just about collecting data. It is about participant safety, team awareness, adaptability, communication and knowing when to push forward and when to pause to ensure that data are collected safely and precisely.

Of all the projects we ran, the SGB study was perhaps one of the most exhilarating. To our knowledge, no one has attempted a SGB at high‐altitude, where sympathetic activity is already elevated. Naturally, this meant the project was equal parts exciting and ambitious, but of course, no experiment unfolds without hiccups. On the first day, we encountered the kind of problem that only field research can deliver: we forgot our 3 L syringe, which is used to calibrate our respiratory pneumotachometer. In a conventional lab, this would be annoying. At 3800 m, far from the nearest fully stocked supply room, it became a team‐based engineering challenge. At one point, Stacey looked at Tymko, both working on separate improvised solutions, and said something to the effect of, ‘You work on yours, I'll work on mine, and we'll see who comes out on top.’ It was less of a troubleshooting session and more of a low‐oxygen episode of MacGyver coupled with blueprint designs drawn up by Heath Robinson. The students were intrigued and they got to watch some late‐night problem solving amongst the PIs in the small workshop the remote research station had available, and to no one's surprise, Stacey's solution came out on top at about 3 a.m., mere hours before testing– a simple configuration of compressing a 20 L Douglas bag through a custom three‐way valve and an analogue spirometer we luckily had. These are the moments trainees remember. Not because everything went perfectly, but because it did not. They watched senior researchers’ problem‐solve calmly, creatively and collaboratively under pressure. That is difficult to teach in a lecture hall.

On one memorable morning, Fernandes was our final participant for the SGB study. Tymko had been sleeping in the laboratory the night prior due to overcrowding, which allowed him to literally roll out of bed and turn on the equipment. That day, however, he overslept. Fernandes gently shook him awake and whispered, ‘Mike, it's time to do science.’ Slightly embarrassed but highly motivated, Tymko scrambled to set up. What followed was both comedy and triumph. Fernandes, determined to perform his own microneurography to obtain a muscle sympathetic nerve signal while also undergoing the ganglion block. He managed to locate a sympathetic nerve signal despite interference from the station's eclectic electrical wiring. All the students at the research station gathered around in excitement and awe. At one point, when the signal deteriorated, Fernandes was trying to determine where the electrical noise was coming from before calmly announcing to the group, ‘Wait… I am the noise.’ He grounded himself, stabilized the recording, and we carefully laid him supine for the block, all without losing the signal. When the SGB took effect, and it was apparent we were successful in this trial, Fernandes, lying motionless with electrodes in place, reached for Tymko's hand and whispered, ‘Mike, we just made history my friend. Niels Secher is jealous right now.’

For Fernandes, this was both a scientific milestone and a tribute to one of his mentors. For us, it captured why we love this work: the thrill of pushing boundaries, the laughter threaded through late‐night problem‐solving, and the curiosity that drives people to ask bold questions in inconvenient places. Maybe one day, one of our students will recreate a similar story and think to themselves ‘Bain (or Smith/Tymko/Stacey/Fernandes) would be jealous right now’. It was a beautiful scientific moment.

Our group also took advantage of the nearby 4000+ m peak of White Mountain for some extra ‘altitude training’ and exercise when time permitted. Near the end of the expedition, we summited as a group to close out the trip. For the PIs, this was one of the highlights: sharing the beauty, difficulty and camaraderie of field research with our graduate students, while reinforcing the importance of teamwork, leadership and looking out for one another. Many students expressed that the meandering hike, while not difficult terrain, was one of the hardest things they have ever done physically, this despite already being acclimatized to 3800 m. The best part of these expeditions, though, is not just the data. It is the relationships. We are not just colleagues; we are now lifelong friends forged through shared hardship, scientific adventure, communal living, occasional equipment failure and watching together an old VHS tape of *A Cold Night's Death* (1973), a movie that was originally filmed at the Barcroft Research Station. Together, we try to balance rigorous research with creating a transformative experience for our students.

That, ultimately, may be the most important outcome of the expedition. Yes, we collected exciting data. Yes, we pushed ambitious physiology into a challenging environment. And yes, we occasionally wondered whether the mountain, the equipment or the altitude would win. But we also trained the next generation of scientists in a way that only field research can. Our HQP gained technical skills, confidence, independence, teamwork, resilience and a deeper appreciation for what it means to do integrative human physiology. And if they also learned that the best scientific ideas sometimes begin in a hot tub at a ski resort, well, that is just part of the training. Upon reading the studies in this special journal article call, it is important to remember that what is published, whether it be related to invasive SGB and autonomic blockades, arterial catheterization and blood vessel responses, respiratory response to exercise, or brain blood flow responses to brain freeze headaches, perhaps only tells a third of the story.

## AUTHOR CONTRIBUTIONS

All authors were responsible for drafting of the manuscript. All authors have read and approved the final version of this manuscript and agree to be accountable for all aspects of the work in ensuring that questions related to the accuracy or integrity of any part of the work are appropriately investigated and resolved. All persons designated as authors qualify for authorship, and all those who qualify for authorship are listed.

## CONFLICT OF INTEREST

None declared.

## GENERATIVE AI STATEMENT

Artificial intelligence tools were used when sourcing references for this manuscript (ChatGPT v5.5).
